# Nitrogen fixation in microbial biotechnology: An annotated selection of World Wide Web sites relevant to the topics in microbial biotechnology

**DOI:** 10.1111/1751-7915.12313

**Published:** 2015-08-11

**Authors:** Lawrence P Wackett

**Affiliations:** Department of Biochemistry, Molecular Biology and Biophysics, BioTechnology Institute, University of MinnesotaSt. Paul, MN, 55108, USA

## Nitrogen fixation by legumes

### http://www.csun.edu/~hcbio027/biotechnology/lec10/lindemann.html

This review provides a good overview of biological nitrogen fixation in the growth of leguminous plants, particularly relating to fertilizer needs for crops.

## Nitrogen fixation in non-legume plants

### http://aob.oxfordjournals.org/content/early/2013/03/08/aob.mct048.full

In addition to plants that form nodules that host nitrogen-fixing bacteria, other bacteria form different associations with plants, and this is currently under intense study to determine if there is significant nitrogen transfer to the plants.

## Leaf bacteria fertilize trees

### http://www.sciencemag.org/content/348/6237/844.summary

It is being proposed that one of the fastest growing trees, poplar, uses nitrogen-fixing bacteria to help them grow more quickly. The report also mentions the potential for bacterial nitrogen-fixers to support the growth of certain evergreen trees.

## Nitrogen fixation in cereal crops

### http://www.producer.com/2014/04/the-search-for-the-holy-grail-nitrogen-fixation-in-cereal-crops/

This article discusses efforts to effectively use microbial nitrogen fixation to boost the growth of cereal crops and require a lesser use of fertilizer, a development that would have very important implications for society.

## Engineering nitrogen fixation in *Escherichia coli*

### https://www.jic.ac.uk/news/2014/10/progress-towards-sustainable-biological-nitrogen-fixation/

This article reports on a research paper describing the expression of the iron-dependent nitrogen fixation enzymes in *E. coli*.

## Origin of biological nitrogen fixation pushed back one billion years

### http://www.oregonlive.com/pacific-northwest-news/index.ssf/2015/02/earlier_life.html

This article presents isotope evidence that biological nitrogen fixation by the molybdenum nitrogenase originated approximately 3.2 billion years ago.

## Microbial nitrogen-fixers feed grasses

### http://cafnrnews.com/2015/06/taking-root/

This news article discusses research demonstrating that a grass receives its total nitrogen needs from bacteria associated with the root surfaces.

## Microbial nitrogen-fixation genes engineered into plant organelles

### https://jwafs.mit.edu/research/projects/2015/engineered-nitrogen-fixation-expression-plant-organelles

This page describes a currently-funded research project to clone and express a re-engineered nitrogen fixation operon from a bacterium into plants.

## Has a start-up solved the nitrogen problem?

### http://www.agweb.com/article/has-a-startup-solved-agricultures-nitrogen-puzzle-NAA-chris-bennett/

This article describes the use of a nitrogen-fixing bacterium, *Gluconacetobacter diazotrophicus*, in a product that is being applied in field trials on oilseed rape, wheat and pasture grass.

## Nitrogen fertilizer damages plant–microbe mutualism

### http://www.laboratoryequipment.com/news/2015/02/nitrogen-fertilizer-slowly-damages-plant-microbe-mutualisms

The main message of this news article is the idea that exposure of rhizobia bacteria to nitrogen fertilizer over some years results in their nitrogen-fixing ability becoming less beneficial to leguminous plants.

## Bacteria tracked feeding nitrogen to plants

### https://www.bnl.gov/newsroom/news.php?a=25582

This news article points out the efficacy of using nitrogen-fixing bacteria to stimulate the growth of grasses and that these organisms are sold as crop inoculants in South America.

## BBC: Nitrogen fixing

### http://www.bbc.co.uk/programmes/b03k21p5

This video provides a good overview of nitrogen fixation by chemical and biological means.

## New developments in biological nitrogen fixation

### http://news.nationalgeographic.com/news/2014/09/140918-soil-bacteria-microbe-farming-technology-ngfood/

This article describes recent research on biological nitrogen fixation and new findings that non-leguminous crops can obtain significant nitrogen from bacterial nitrogen fixation.

## Ceres: Nitrogen fixation products in biotechnology

### http://www.ceresorganics.in/products/biotechnology-based.html

Ceres is a commercial entity that sells various formulations of nitrogen-fixing microbial fertilizer mixtures.

## Non-symbiotic nitrogen fixation

### http://www.biocyclopedia.com/index/biotechnology/plant_biotechnology/biological_nitrogen_fixation/biotech_non-symbiotic_n2_fixation.php

The Biocyclopedia lists many non-symbiotic nitrogen-fixing bacteria and contains links to other relevant information.

## Free-living bacteria increase soil nitrogen

### http://www.clw.csiro.au/publications/farming_ahead/2006/KYM_NSNF%20final_%20FEB_2006.pdf

This web brochure describes the contribution of non-leguminous nitrogen fixation to agriculture in Australia.

